# A STAT3 of Addiction: Adipose Tissue, Adipocytokine Signalling and STAT3 as Mediators of Metabolic Remodelling in the Tumour Microenvironment

**DOI:** 10.3390/cells9041043

**Published:** 2020-04-22

**Authors:** Rose Kadye, Mihlali Stoffels, Sidne Fanucci, Siso Mbanxa, Earl Prinsloo

**Affiliations:** Biotechnology Innovation Centre, PO Box 94, Rhodes University, Grahamstown 6140, South Africa; g17S4194@campus.ru.ac.za (M.S.); g13F0202@campus.ru.ac.za (S.F.); g15M5263@campus.ru.ac.za (S.M.)

**Keywords:** STAT3, mitochondrial STAT3, adipose tissue, cancer, oncogenesis, inflammation, adipocytokine signalling

## Abstract

Metabolic remodelling of the tumour microenvironment is a major mechanism by which cancer cells survive and resist treatment. The pro-oncogenic inflammatory cascade released by adipose tissue promotes oncogenic transformation, proliferation, angiogenesis, metastasis and evasion of apoptosis. STAT3 has emerged as an important mediator of metabolic remodelling. As a downstream effector of adipocytokines and cytokines, its canonical and non-canonical activities affect mitochondrial functioning and cancer metabolism. In this review, we examine the central role played by the crosstalk between the transcriptional and mitochondrial roles of STAT3 to promote survival and further oncogenesis within the tumour microenvironment with a particular focus on adipose-breast cancer interactions.

## 1. Introduction

The remodelling of cancer cells in a tumour environment is a need to ensure survival and proliferation in response to extrinsic and intrinsic signals. The interaction of cancer cells with the stroma modulates the microenvironment to be more abetting of oncogenesis thereby: stimulating tumour growth and proliferation, increasing resistance to growth-inhibitory signals and evasion of apoptosis, promoting vascularization, migration and tissue invasion. To enable this it is key to promote the remodelling of metabolism across the heterogeneous tumour population bone marrow X-linked (BMX) nonreceptor tyrosine kinase [1,2,3]. Adipose tissue in obesity provides a dysregulated low grade inflamed macroenvironment offering a cytokine and chemokine flood that plays a major role in tumour neoplastic development [4,5,6].

Signal transducers and activators of transcription (STATs) are transcription factors associated with multiple essential cellular processes, including the key hallmarks of oncogenic initiation: proliferation, survival and angiogenesis. As an oncogene, typically overexpressed in cancers, STAT3 regulates the expression of numerous downstream oncogenes including itself. Beyond canonical tyrosine 705 phosphorylation in Janus kinase-STAT3 (JAK-STAT3) signalling, it directs and promotes cancer growth and metastasis through non-canonical signaling by serine phosphorylation at position 727 as well as in its unphosphorylated state [7,8,9]. Evidence suggests that inflammatory signals from the macroenvironment and the tumour microenvironment promotes and sustains oncogenesis through the production and release of pro-survival factors such as interleukin-6 (IL-6). Thus effectively creating an endless feedback loop of paracrine and autocrine signalling to kickstart and maintain metabolic reprogramming while halting apoptosis [10,11,12]. The inflammatory response, while typically providing a coordinated line of defence, atypically acts to promote and enhance tumour initiation and progression [13]. Adipose tissue is by and large recognized as an endocrine organ. Metabolic imbalance resulting in accumulated adipose tissue (obesity) provides a binary “always on switch” of low-grade inflammation and sustained release of pro-inflammatory cytokines that activate phosphorylated and non-phosphorylated STAT3 signalling [4]. While not discussed in detail in this review it should be noted that unphosphorylated STAT3 signalling appears to be reliant on IL-6 signalling and appears to further promote and and sustain an addiction to gp130-linked cytokine signaling [14,15]. Furthermore, STAT3 interactions with transcription factor super-complexes (super-enhancers) are known to enhance oncogenic signalling pathways [16,17,18]. Here, we posit the inter-connected transcriptional and mitochondrial roles of STAT3 as a mechanistic linchpin between cellular transformation and eventual cancer progression using adipose tissue as a case study.

## 2. Cancer: An Interplay of Canonical and Non-Canonical STAT3 Signalling

**Figure 1:**
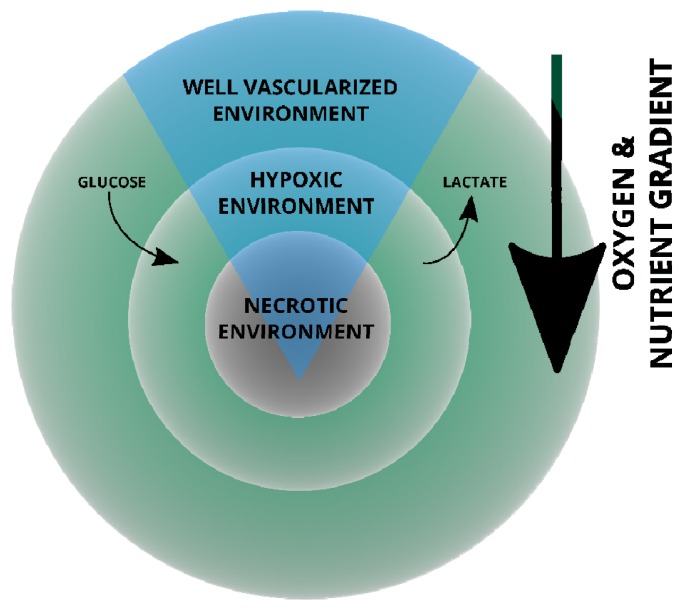
Illustrated cross-section of a human breast cancer tumour. This schematic represents an oversimplified view of the gradients that exist from the well-vascularized environment that forces cellular remodelling and nutrient exchange. Deprivation along the gradient invariably results in necrosis [17,19].

The heterogeneity of tumours is illustrated by the schematic of a breast cancer tumour in Figure 1, in which aerobic, well oxygenated tumour cells surround blood vessels whereas poorly oxygenated hypoxic regions are located further away from the blood supply [17].

This allows for glycolytic hypoxic and aerobic regions of the tumour and the cells therein to exchange metabolites; hypoxic cells generate lactate which is converted to pyruvate used in oxidative phosphorylation (OXPHOS) to generate ATP by aerobic cancer cells, leaving more glucose for hypoxic cancer cell metabolism in “metabolic symbiosis” [20,21,22,23,24]. Glucose uptake following upregulation of hypoxia inducible factor 1 (HIF-1) results in the upregulation of glucose transporter 1 (GLUT-1) expression [25]. The glucose is converted to pyruvate which in turn is converted to lactic acid. The production of lactic acid within the hypoxic cell by c-MYC upregulated lactate dehydrogenase (LDH-α), is responsible for the conversion of pyruvate into lactate [26]. This decreases the pH in the environment when lactic acid is transported out of the cell. The lower pH further promotes tumour invasion and survival through the modification of the microenvironment yielding it toxic to immune cells [26]. Contrary to normal cells that only resort to anaerobic glycolysis when oxygen is limiting, most cancer cells depend on the Warburg effect, a glycolytic shortcut, to derive energy in order to sustain rampant proliferation, even in normoxia and hyperoxia [22,27,28,29]. In addition to glycolysis, the bioenergetic needs of tumour cells are also met through alternative routes such as fatty acid oxidation (reviewed in [30]). Despite the decrease in OXPHOS and ATP production, the Warburg shunt produces glycolytic precursors for the de novo biosynthesis of carbohydrates, proteins and fats; building blocks required by proliferating cells [29,31]. Similarly, tricarboxylic acid (TCA) cycle intermediate pools, to be used as biosynthetic carbon sources are maintained [28]. Tumour cells exploit the metabolic capacity of surrounding cells in the microenvironment; reprogramming nutrient acquisition metabolic pathways to meet bioenergetic and biosynthetic needs. This flexibility in cancer metabolism not only allows rapid tumour cell proliferation, but also renders tumour cells the ability to survive.

As a signal transducer and a transcriptional activator, STAT3 shuttles between the cell membrane and the nucleus [32,33,34]. It also localizes in other sub-cellular compartments most notably in the mitochondria in normal and aberrant cellular phenotypes [1,14,35]. Here, we discuss its role in Warburg effect and maintenance of a cancerous phenotype.

### 2.1. Canonical STAT3 Signalling and the Warburg Effect

**Figure 2:**
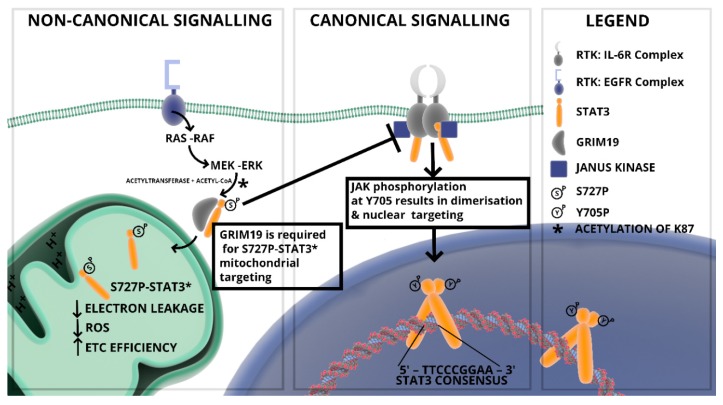
Canonical and non-canonical STAT3 signalling. The phosphorylation of STAT3 at residues Y705 and S727 in canonical and non-canonical signalling (respectively) dictate cellular distribution and activity.

Multiple signalling pathways converge through STAT3 signalling which in turn determines the post-translational modification/activation and subcellular localization and ultimately the function of STAT3 as illustrated in Figure 2 [36].

The STAT3 signalling pathway is canonically activated through tyrosine phosphorylation and it is constitutively activated in a majority of tumours often leading to a STAT3 addiction [36]. The canonical activity of STAT3 is mainly dependent on the Janus kinase (JAK) phosphorylation at tyrosine 705 (Y705) [37]. The canonically activated pY705 STAT3 is nuclear targeted and associates with the promoters of early response genes to: promote proliferation, enhance tissue invasion and metastasis (e.g., cyclin D1 and matrix metalloproteinase-2/-9 in breast cancer) [38,39,40,41], activate terminal differentiation and growth arrest, promote evasion of the immune response by suppressing apoptosis [42], promoting angiogenesis, as well as modification of cellular energy metabolism and mitochondrial activity [43,44]. The activation may induce lysosome-mediated apoptosis depending on cell type and conditions or stimulation [45]. As such, dysregulated STAT3 activation is positively correlated with a majority of cancers; regulating functional pleiotropic responses ranging from cell proliferation to angiogenesis to metastasis [8,46,47]. Constitutively activated nuclear STAT3 induces a metabolic switch toward aerobic glycolysis through transcriptional activation of HIF-1α and down-regulation of mitochondrial activities [48,49,50,51]. While traditionally associated with activation through the gp130 receptor family, the signals that trigger phosphorylation of tyrosine 705 range from extracellular cytokines, through to hormones from the adipose tissue, growth factors, receptors, oncogenes [36,52,53,54,55], or constitutive activation by reactive oxygen species (ROS) in aberrant signalling [8,37,56] highlighted in Table 1. Because the activation of STAT3 occurs in both cancer and stromal cells, it allows for crosstalk of STAT3 signals.

Signal transducer and activator of transcription 3 (STAT3) activation is counterbalanced by three main groups of negative regulators; phosphatases, Protein Inhibitor of Activated STAT (PIAS) proteins and Suppressor of Cytokine Signalling (SOCS) proteins [70,71,72,73,74]. While phosphatases up-regulated in cancer counteract the JAK-mediated phosphorylation or terminate the activation of STAT3 [70,71,75], PIAS proteins which are upregulated in breast cancer, inhibit STAT3 DNA binding activity [73,76], and SOCS proteins, that inhibit JAK-STAT3 signalling through negative feedback, are downregulated in breast cancer [77]. While the negative regulation actively modulates STAT3 activity, when compromised, it acts to synergistically enhance aberrant stimulation and constitutive activation of STAT3.

### 2.2. Mitochondrial STAT3: Inside the Engine Core

Despite lacking a mitochondrial localization signal, acetylated, serine and tyrosine phosphorylated STAT3 have been detected in mitochondria mostly of cancer cells [78]. Nonetheless, post-translational modifications activated downstream of cytokines, growth factors and oncogenes shape the sub-cellular localization and activities of STAT3 both in cancer and normal cells. In addition to Y705P, STAT3 can also undergo several other post-translational modifications such as phosphorylation on S727 in oncogenic transformation [79,80], lysine acetylation or methylation and cysteine oxidation or glutathionylation in starved cancer cells stimulated with serum or insulin, and in cardiomyocytes as part of reduction-oxidation (REDOX) regulation [81,82,83]. Serine 727 phosphorylated STAT3 (pS727 STAT3) exhibits distinct mitochondrial localization and non-transcriptional function through the modulation of the activity of the electron transport chain (ETC) and the mitochondrial permeability transition pores (MPTP) [1,84]. The serine 727 phosphorylation event is essential for mitochondrial localization and activity; this occurs through the mitogen-activated protein (MAP)/extracellular signal–regulated kinase (ERK/MEK) signalling axis [80,81]. In addition, pS727 STAT3 is also reported to negatively regulate pY705 STAT3, thereby inhibiting dimer formation; shifting the pool available for DNA binding [73]. The components of the full regulatory signalling pathway that controls mitochondrial STAT3 targeting is slowly being uncovered. Strong evidence point to acetylation of STAT3 at lysine 87 residue by CBP/p300 histone acetyltransferases downstream of cytokines, growth factors and Ras signalling proteins [85,86], resulting in a GRIM-19-dependent mitochondrial translocation where it modulates pyruvate metabolism, the activities of ETC complexes and ROS production [83,87,88] (Figure 2). Evidence suggests that phosphorylation at the S727 residue is important for interaction with GRIM-19 [89]. Mitochondrial STAT3 (pS727 STAT3/mitoSTAT3) interacts with complexes of the ETC to form stable respiratory chain super-complexes that preserves the optimal transfer of electrons within individual complexes and minimizes electron leakage [1,81,90]; consequentially this increases mitochondrial membrane potential (MMP) and ATP synthesis while ROS levels decrease thereby promoting cell survival [83,90,91]. Therefore, mitoSTAT3’s augmentation of the activities of the ETC complexes is pro-survival related, as it enhances cell fitness under the stressful conditions associated with ischemia-reperfusion injuries and Ras-dependent oncogenic transformation, suggesting that mitoSTAT3 promotes oncogenic transformation by modulation of mitochondrial activity [92]. As such, mitoSTAT3 thus supports oncogenic cellular transformation associated with aberrant oncogenic Ras signalling reported to wreak havoc in approximately a third of all cancers, resulting in uncontrolled proliferation and silencing of the cell death machinery [93,94].

The mitochondrial localization of STAT3 and influence on ATP generation efficiency and hence energy supplies contributes to cell development, proliferation and survival. This has consequences in normal and aberrant cell development, for example, mitoSTAT3 is required for neurite outgrowth following neurotrophic factor stimulation [95]. Conversely, mitoSTAT3 activity protects the chronic lymphocytic leukaemia (CLL) phenotype from oxidative damage [60,96]. Transformed cells have the activities of gamma-glutathione optimized by mitoSTAT3 to restrain ROS levels thus preventing death. Interestingly, mitoSTAT3 has also been associated with increased ROS levels potentially as a consequence of the variability of downstream activation targets in different cellular phenotypes [78,97,98]. Therefore, mitoSTAT3 aids both normal and cancer cells to maintain mitochondrial homeostasis and integrity to prevent processes that lead to cell death. However, during the different stages of cancer development (initiation, promotion, progression or apoptotic induction) and environmental location within a tumour mass cross-section (see Figure 1), the mitochondrial respiration requirements and metabolic states of cells determines the specific varying effect of both canonical and non-canonical STAT3 on mitochondrial membrane potential and concomitant production of ROS. Calcium efflux into the cytoplasm is strongly associated with apoptosis and mitoSTAT3 plays a crucial role in Ca^2+^ homeostasis by sustaining the mitochondrial membrane potential gradient used by mitochondrial calcium uniporter for the mitochondrial uptake of Ca^2+^ [84]. Mitochondrial STAT3 also contribute to the maintenance of Ca^2+^ homeostasis through inhibition from opening of the nonselective Ca^2+^-dependent MPTP [84]. Overall, although nuclear and mitoSTAT3 mechanisms may be different, the cellular roles complement one another with regards to regulating ROS, preventing apoptosis or maintaining mitochondrial integrity.

### 2.3. Metabolic Remodelling through STAT3 Stimulation

Direct mitochondrial metabolic remodeling through the activity of STAT3 was shown via the serine phosphorylation of mitochondrial STAT3; a RAS/MEK/ERK mediated transformation in a mouse model through the modulation of aerobic glycolysis and ETC activity [79,91]. The nuclear influence of canonically activated STAT3 on metabolism was shown to promote a shift towards glycolysis through HIF-1α-induced pyruvate kinase M2 isoform (PKM2) chronic activation of STAT3; this in turn activates HIF-1α; this positive feedback loop supports proliferative and pro-survival phenotypes [48,49,99]. In addition, nuclear phosphorylated Y705 STAT3 also downregulates mitochondrial activities through transcriptional regulation of ETC complex proteins with direct effects on mitochondrial respiration, ROS levels and apoptosis [93]. The switch toward aerobic glycolysis is essential for rapid tumour cell proliferation; this renders tumour populations flexible to limiting environments hence many different tumours persist through a STAT3 addiction [49,99]. In normal liver tissue, STAT3 suppresses the expression of glucose-6-phosphatase resulting in suppression of gluconeogenesis by increasing hepatic glucose production, and as such is crucial in liver glucose homeostasis [100]. In muscle, IL-6 promotes glycogenolysis and lipolysis to increase the availability of glucose and lipids [101]. In the pancreatic cells, IL-6 stimulation leads to insulin release which shifting the balance from glucagon-dependent catabolic pathways to insulin-dependent anabolic pathways [101].

A positive energy balance is the main reason surrounding the engorgement of adipocyte cells during obesity. This is due to the excess energy being stored as triacylglycerols within adipose tissues, which can cause metabolic dysfunction within cells [102]. High levels of circulating leptin have been associated with obesity due to its secretion by adipose tissue. These levels have been linked to increased cancer risk, particularly in colon and breast cancer [102].

**Figure 3:**
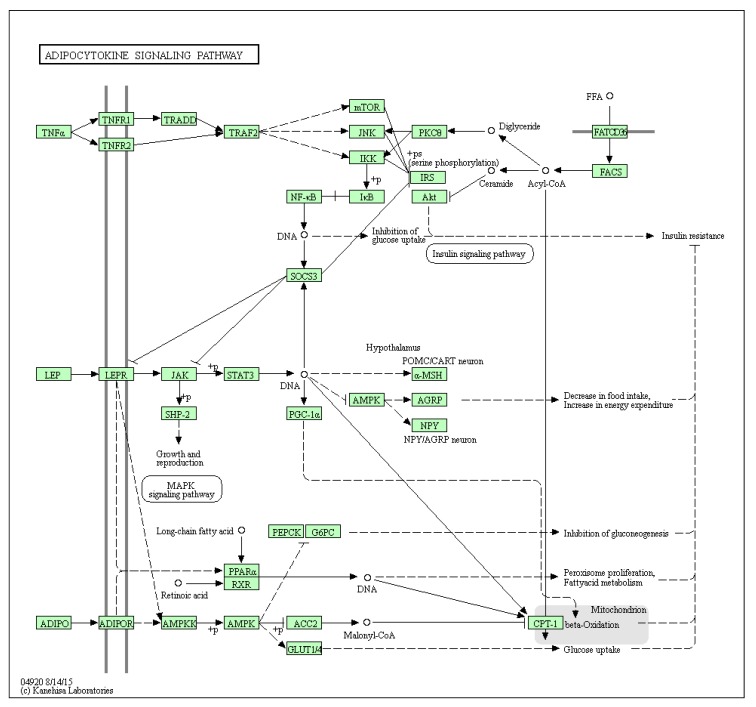
The adipocytokine signaling pathway in humans (KEGG hsa04920). Image reused with permission [121,122,123].

Obesity related adipose tissue dysfunction exhibit abnormal signalling pathways that have the ability to change the metabolic profile of the cell and promote tumorigenesis (reviewed in [102]. High levels of leptin, for example, has been linked to the activation of the JAK-STAT pathway through the LEPR receptor in the adipocytokine signalling pathway (Figure 3).

Adipocytes may provide alternative energy sources for cancer in the form of lipids; Dirat and co-workers have shown how breast cancer-adipocyte co-culture results in delipidation of the adipocytes and an enhanced invasive phenotype in the cancer [103]. Interestingly, fatty acid oxidation (FAO) was linked to decreased proliferative potential in ER positive breast tumours; the authors concede that ER negative tumours are of course considered more proliferative [104]. It should be noted that the use of lipids as an energy source is up for debate as they may equally serve as sources for biosynthesis or act as oncogenic lipid signalling sources [105,106]. When considering the leptin activation (see Figure 3—Adipocytokine signaling pathway) of the JAK/STAT3 pathway, STAT3 directly upregulates FAO through transcriptional activation of carnitine palmitoyltransferase 1B (CPT1B) in breast cancer stem cells; promoting stemness and chemoresistance [107]. Peritumoral adipocytes show a primed or activated phenotype (termed cancer associated adipocytes) which were characterized by increased expression of matrix metalloproteinase-11 and most notably IL-6 [103]. Breast cancer progression is promoted further by leptin stimulated STAT3 mediated FAO in CD8^+^ T effector cells [108]. Recently, Yu et al. [109] reported that ovarian cancer progression can be driven by the proinflammatory cytokine IL-17A released by T helper 17 cells (Th17) that promotes fatty acid uptake in adipose rich environments through STAT3 and Fatty acid-binding protein 4 (FABP4). These cells are likely recruited and stimulated through obesity related inflammation.

By extension this has large implications for STAT3 mediated signalling in ER positive and negative breast cancers in the potential promotion of invasion, metastasis and usage of lipids for metabolic maintenance of cancer promoting/tumour initiating populations of cancer stem cells. Considering the stem cell origin of breast cancer [110] the role of STAT3 in normal stem cells cannot be overstated due to the role of the JAK-STAT3 pathway in cellular transformation [47,111]. Differentiation of murine mammary stem cells has been shown to require STAT3 activity and furthermore it is necessary for the maintenance of proliferation of luminal ductal progenitors [112]. The potency of mammary stem cells are still up for debate yet it should be noted that populations have been marked as LGR5^+^, a critical stem cell marker [113,114,115]. It should be noted that human mammary stem cells self-renewal is largely driven by collaborative signaling through Notch, Wnt and Hedgehog signaling [116]; these signaling networks are the drivers of self-renewal of LGR5^+^ breast cancer stem cells [117] as well (reviewed by Yang et al. [118]). LGR5 expression has been linked to STAT3 activity through interplay with IκB-kinase alpha (IKKα) in basal cell carcinoma [119]. While it may be tempting to speculate on the role of STAT3 in LGR5 expression in mammary stem cells and breast cancer stem cell development given the interplay between NFKB and STAT3 signalling [120], this however requires further experimental evidence.

## 3. REDOX Signalling in Cancer: Does STAT3 Maintain the Balance?

The intrinsic generation of ROS, a by-product of mitochondrial respiration, is regulated by STAT3; high ROS levels greatly affect cancers and STAT3 addiction [49,93,124]. The metabolic shift during oncogenic transformation is characterized by an increase in production of mitochondrion-derived ROS [125,126], and, as such, it is critical for proliferation and survival to maintain REDOX balance [127]. These elevated ROS levels can promote tumourigenesis through destabilizing the genome and increasing ROS dependence in signalling pathways [127,128,129]. Despite cancer cells producing the bulk of ATP through the Warburg effect, the mitochondria are still active and contribute to ROS production through OXPHOS [40,41,130,131], and maintenance of Ca^2+^ homeostasis [132,133,134,135,136,137]. Myeloid progenitors produce a much higher ROS than leukocytes produced in mammalian hematopoietic systems. Since leukemic cells experience more exposure to oxidative stress intrinsically, this makes them more vulnerable to oxidative stress. Above basal level increase in oxidative stress in hematopoietic progenitor cells can cause advanced differentiation through the c-Jun *N*-terminal kinase (JNK) signalling pathway; further indicating that the leukaemia phenotype has a correlation with ROS levels [138,139]. In DNA bases of breast cells, purine and pyrimidine can be oxidized by ROS, forming 8-hydroxy-2′-deoxyguanosine (8-OH-dG), which is a breast cancer tissue biomarker for DNA damaged by oxidative stress. It was found that the oxidized form of DNA base (8-OH-dG) was present in lower levels than those found during the early stages of carcinogenesis [140,141]. Whereas low ROS levels play a key role in normal signalling by regulating REDOX signalling pathways, high levels induce permeability of the mitochondrial membrane leading to both the arrest of biosynthetic pathways and mitochondrial induced cell death through apoptosis [129,142,143]. Moreover, oxidative stress as a function of high ROS will disrupt the regulatory control of DNA methylation and methylation patterns [144,145]. High ROS levels therefore results in: (i) site-specific DNA hyper-methylation culminating in gene silencing, and (ii) global hypo-methylation that allows for the expression of usually methylated genes.

**Figure 4:**
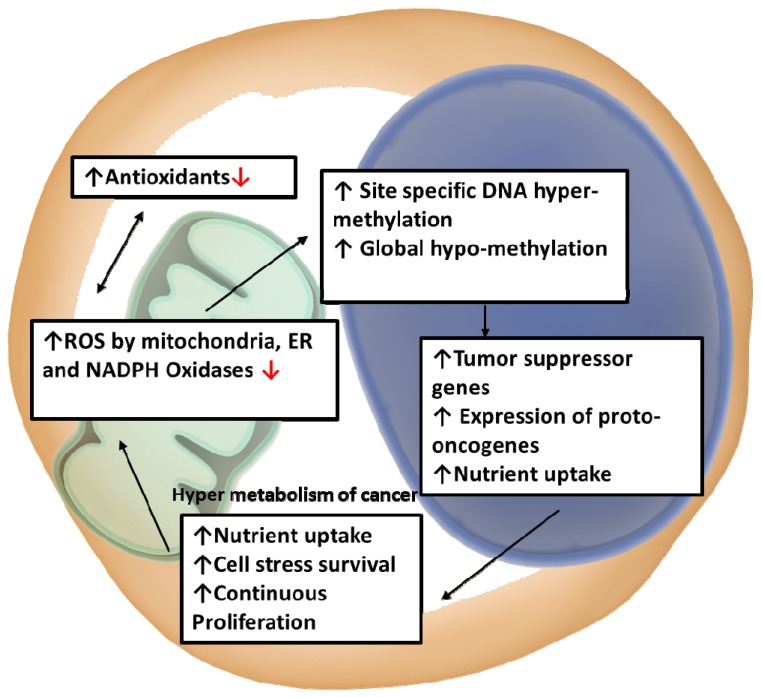
Schematic illustration of REDOX signalling supports oncogenic ROS, metabolic remodelling, evasion of apoptosis and proliferation.

For example, normally, phosphorylated STAT3 is negatively regulated by Src homology 2 (SH2) domain-containing protein tyrosine phosphatase 1 (SHP-1) [146]. However, high ROS levels result in epigenetic silencing of SHP-1 through hyper-methylation, leading to constitutive activation of STAT3 (Figure 4) [146]. As such, during early events of carcinogenesis i.e., neoplasia progression and metastasis, global hypo-methylation and regional hyper-methylation lead to the suppression of tumour suppressor genes (TSGs) and the expression of proto-oncogenes [144]. The hyper-metabolism of cancer cells allows for continuous proliferation and survival through the uptake of abundant nutrients, resulting in high ROS generation from mitochondria, endoplasmic reticulum and NADPH oxidases offset by a highly augmented antioxidant activity [80,89]. However, if these high ROS levels are not regulated, they can leave cancer cells susceptible to oxidative stress-induced cell death [147]. Relative to normal phenotypes transformed cells may exhibit variable levels of ROS depending on the balance between ROS production and scavenging; even in the noise of high internal levels, ROS dependent signalling systems are localized to point sources to allow for a pro-survival phenotype [148,149]. Mitochondrial STAT3 (mitoSTAT3) limits mitochondrial and cellular ROS production but appears to promote ETC activity; the mechanism remains unclear [89,92]. While the function of STAT3 is also regulated by post-translational oxidation, ROS may also be important for controlling mitoSTAT3 levels [80,150]. Hypoxic zones within solid breast cancer tumours are associated with elevated mitochondrial ROS production, this appears to be linked to the modulation of the ETC by mitoSTAT3; allowing fine control of ROS production [20].

In adipose tissue, the differentiation of adipocytes is dependent on the shift in REDOX balance [151]. Our work in the 3T3-L1 mouse model showed a strong correlation between initiation of adipogenesis and the mitochondrial localization changes of S727 STAT3; a REDOX burst in observed early in adipogenesis as STAT3 translocates to the cytoplasm from the mitochondria [99]. Seemingly, a requirement for normal differentiation. Compared early adipocytes, mature-insulin-sensitive adipocytes establish a new and higher REDOX operating equilibrium [152,153]. A shift in REDOX beyond this may disrupt this balance resulting in the adipocyte nutritional overload and therefore result in an increase in oxidative stress and an inflammatory promoting environment. In obesity a progressive increase in ROS (H_2_O_2_) and its counteracting reductants reflect this [154].

## 4. STAT3 as a Regulatory Buffer of Apoptosis and Autophagy

Signal transducers and activators of transcription 3 (STAT3) are important in buffering the abnormal activation and deregulation of survival, apoptosis and autophagy pathways within the tumour microenvironment [155,156,157,158]. Apoptosis is activated by both intrinsic (including DNA damage, oxidative stress or uncontrolled proliferation) and extrinsic signals through two core pathways [159]. The extrinsic pathway involves activation of Fas and tumour necrosis factor (TNF) receptors whereas, the intrinsic pathway involves the MMP’s and the MPTP’S mitochondrial to cytoplasmic leakage of pro-apoptotic factors (e.g., cytochrome c and apoptosis-inducing factor) [160,161]. Both pathways result in the activation of caspase-related cell death machinery [162]. Despite typically evading apoptosis, cancer cells are ‘primed for apoptosis’ relative to normal cells [163,164,165]. Numerous inhibitors of apoptotic pathways like Bcl-2 are overexpressed in tumours, whereas pro-apoptotic proteins like BAX are downregulated [134,166,167]. These apoptotic defects raises the threshold needed for cell death and allow tumour cells to resist traditional chemo- and radiotherapies [166,167]. Canonically activated STAT3 transcriptionally activates genes critical for regulating cell survival and proliferation (e.g., c-Myc, cyclin D1, and Bcl-family proteins) [168,169]. In chronic lymphocytic leukaemia (CLL) cells, although both pS727 STAT3 and pY705 STAT3 activate the transcription of the same repertoire of genes, constitutively activated pS727 STAT3 not only activated anti-apoptotic genes, but also shows low-affinity binding to the caspase-3 promoter [169]. However, when overexpressed, STAT3 promoted apoptosis through induction of the expression of caspase-3 [169]. Furthermore, mitoSTAT3 contribute to anti-apoptotic functions in tumour cells by increasing the MMP and inhibiting the opening of the MPTP to release Ca^2+^ and cytochrome c into cytosol which would lead to intrinsic apoptosis as demonstrated in human esophageal squamous cell carcinoma (ESCC) [170,171]. Therefore, the role of STAT3 in apoptosis depends not only on the localization of STAT3, but its levels.

Dysregulation of autophagy, while a mediator of homeostasis, is associated with cancer, neurodegeneration, heart and liver diseases [172,173,174,175]. Studies have indicated that autophagy is activated as a result of adverse stress such as heat-shock, hypoxia, redox stress and mitochondrial damage [176,177,178,179]. Signal transducer and activator of transcription 3 (STAT3) has been shown to inhibit autophagy by interfering with the cytoplasmic protein kinase R (PKR) interaction with eukaryotic translation initiation factor 2-alpha kinase 2 (EIF2AK2) via the STAT3 SH2 domain [180,181]. Inhibition of STAT3 SH2 mediated phosphorylation increases the pool of uncomplexed PKR and resulting in increases in basal autophagy. Interestingly in breast cancer, autophagy inhibition is linked with constitutive STAT3 activity [158]; this could be linked to nuclear accumulation of unphosphorylated STAT3 [15]. Inversely, mitoSTAT3 appears to promote autophagy in pancreatic cancer cells [182].

## 5. Adipose Tissue: An Inflammatory Macroenvironment Stimulating the Tumour Microenvironment through STAT3

**Figure 5:**
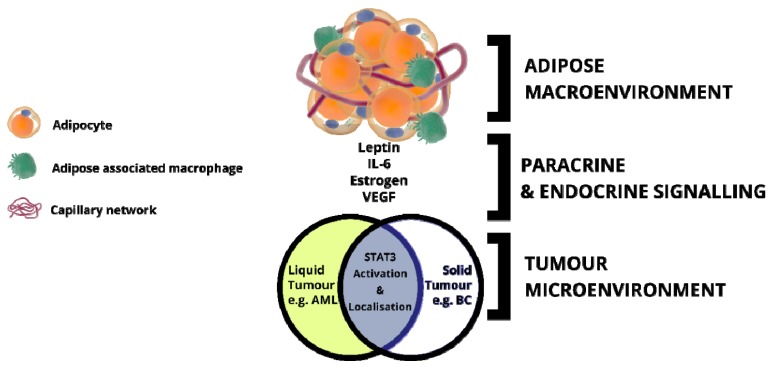
The secretion and releases of adipocytokines and growth factors/cytokines by the obese adipose tissue tumour microenvironment. Tumours in obese adipose tissue are characterized by an environment of low-level chronic inflammation known to upregulate growth factors and adipocytokines that promote cancer development and progression.

**Figure 6:**
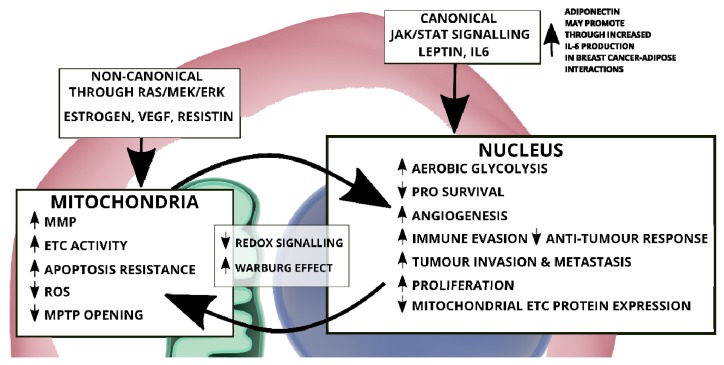
Schematic illustration of how the canonical and mitochondrial roles of STAT3 supports characteristics associated with cancer; ROS, metabolic remodelling, evasion of apoptosis, proliferation.

The growth of tumours relies on mutual interactions with components of the microenvironment comprised of inflammatory cells and cells of hematopoietic and mesenchymal origin that mediate inflammation, angiogenesis and desmoplasia, respectively. One such environment associated in multiple cancers is adipose tissue, an endocrine organ, traditionally associated with maintenance of metabolic homeostasis; imbalance in this results in obesity [183]. Obesity is classically marked by chronic inflammation and altered adipocytokine secretion profiles [183,184,185]. As such, obesity is a well-recognized factor in multiple cancers as a pro-inflammatory, pro-oncogenic macroenvironment that influences tumour initiation growth and development [186]. Firstly, adipocytes and associated inflammatory cells (notably adipose tissue-associated macrophages) secrete various factors such as hormones, adipocytokines, cytokines or growth factors, that mediate systematic effects on the tumour microenvironment and cancer growth [4,187] (Figure 5 and Figure 6). Secondly, the adipocyte to cancer cell-cell contact initiates reprogramming of the adipocytes into cancer associated adipocytes with upregulated factors that solicit metabolic substances (e.g., lipids) from the adipose tissue, further accelerating oncogenesis [13,188]. Adipose tissue-associated macrophages (ATMs) population sizes vary according to the metabolic state with large numbers associated with obese tissues, contributing further to the release of pro-inflammatory cytokines depending on the phenotype; the increased population of the proinflammatory ATM phenotype has been linked to the metabolic activation by free fatty acids through toll-like receptors [189,190]. This proinflammatory phenotype (dubbed M1) therefore feeds back to the mesenchymal stromal/stem and preadipocyte populations to initiate adipogenesis and further maintain the chronic low-grade inflammatory phenotype associated with obese white adipose tissue [191,192]. Changes such as increased mechanical and oxidative stress as well as hypoxic conditions occur within the adipose tissue microenvironment during the process of adipose tissue expansion. It is suggested that these changes induce apoptosis in adipocytes which in turn initiate an influx of alternatively activated (M2) macrophages into the microenvironment [193]. Although considered to have an anti-inflammatory phenotype, M2 macrophages play an essential role in tumor progression and metastasis through suppression of immunity and promoting angiogenesis and matrix remodeling [194]. This complex profile of cells, cytokines, chemokines or growth factors, are therefore responsible for establishing and maintaining an environmental niche that promotes initiation and progression with strong correlations in both liquid and solid tumour types [195,196,197].

Inflammatory signalling pathways involving a plethora of mediators (prostaglandins, cytokines and chemokines), contribute to neoplastic growth and vascularization to sustain and promote cancer growth and development [11]. Normally the inflammatory response is limited by apoptosis and ceases. However, when dysregulated as is the case with chronic inflammation, inflammation results in neoplasia, a process that requires evasion of apoptosis, uncontrolled proliferation, tissue invasion, metastasis and angiogenesis [198]. As such, inflammation in cancer not only promotes cancer cell growth but leads to amelioration of apoptotic signals. This ability of the inflammatory response system to produce and release pro-survival factors allows cells to survive in toxic environments by blocking apoptosis [5]. Furthermore, inflammatory responses can have detrimental effects in cellular apoptotic deletion cancer therapy. Some of the important pro-inflammatory cytokines are of the interleukin-6 (IL-6) family, which communicates the tissues metabolic statuses through activation of the principal downstream effector STAT3 [93,199]. Inflammation and STAT3 signalling in adipose tissue, therefore, work together to create an environment that promotes tumour proliferation and survival through metabolic reprogramming in breast cancer.

As the bulk supporting tissue of breast cancer, IL-6 and leptin release by white adipose tissue has been correlated with paracrine activation of STAT3 (canonical) thereby driving metastatic process [107,155,196,200,201]. In fact, WAT has been associated with decreasing the efficacy of antiproliferative therapeutics in breast cancer [202]. Figure 4 and Figure 5 describe generic models of how the macroenvironment of bulk adipose promotes and drives the development of oncogenesis through endocrine and paracrine signalling. As the cytokines/adipocytokines [92,196,200,203,204] act as stimulatory activators of both canonical and non-canonical STAT3 signalling this drives multiple processes (described in preceding sections) that rely on nuclear and mitochondrial interplay that result in promoting STAT3 addiction and cancer.

**Figure 7:**
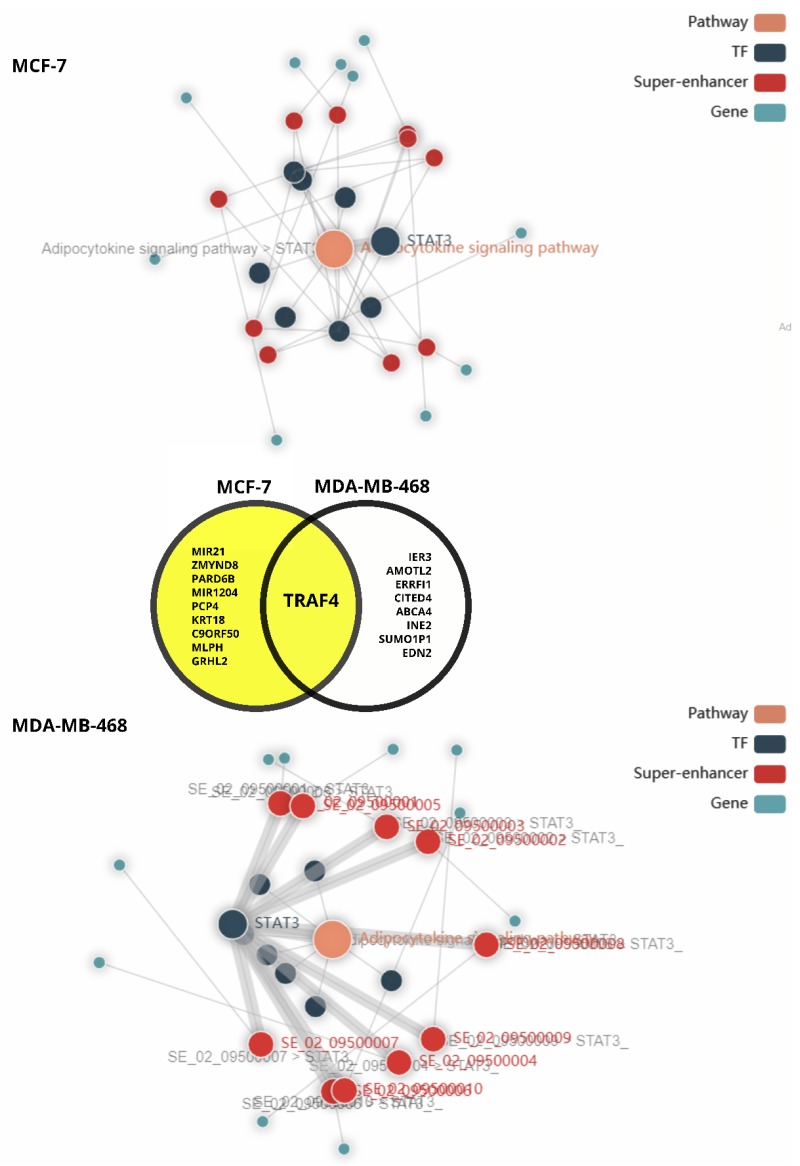
Comparative downstream pathway analysis of the effect of the adipocytokine pathway in breast cancer using the SEanalysis web tool [224,225] for super enhancer associated regulatory analysis. STAT3 was used as a search query for downstream pathway analysis of mammary gland cancers, MCF7 and MDA-MB-468, the adipocytokine signaling pathway was enriched in the mammary gland tissue analysis.

Interestingly, downstream pathway analysis (Figure 7) reveals a difference in STAT3 transcriptional influence of the adipocytokine signaling pathway (Figure 3) in mammary gland derived cancer models, MCF-7 and MDA-MB-468; the latter generally considered a metastatic model. While, the adipocytokine signaling pathway was enriched and predicted to be linked to the expression of multiple genes associated with super-enhancers in the MCF-7 model, STAT3 was not predicted to be associated with any of the super-enhancers in MCF7 compared to the MDA-MB-468 triple negative model; STAT3 was associated with all. Among the top scoring genes associated with the network pathway analysis in MDA-MB-468, unsurprisingly, most genes were associated with promotion of invasion, survival and aggressiveness [205,206,207,208,209,210,211,212,213] relative to those associated with the entire pathway activation in MCF-7 associated with inhibition of tumour suppression and [214,215], promotion of tumourigenesis, migration and survival [216,217,218,219,220,221,222,223].

Apart from STAT3, genes associated with super-enhancers in the pathway include: Retinoid X Receptor Gamma (RXRG), Nuclear factor NF-kappa-B p105 subunit (NFKB1), retinoid X receptor beta (RXRB), retinoid x receptor alpha (RXRA), nuclear factor NF-kappa-B p65 subunit (RELA), NFKB Inhibitor Alpha (NFKBIA) and Peroxisome Proliferator Activated Receptor Alpha (PPARA). Commonly expressed (see Figure 7), the tumor necrosis factor receptor-associated factor 4 (TRAF4) was shown to be an intermediate in the activation of NFKB [226]. Seemingly, there is substantial crosstalk between the NFKB and STAT3 pathways in cancer (reviewed by [120,227]). Furthermore, it appears that IL-6 stimulation leads to accumulation and interaction of unactivated/unphosphorylated STAT3 and NFKB in the nucleus to switch on a subset of cytokine downstream genes; notably IL-6 itself [15]. This likely drives autocrine signaling. Interestingly, synergistic pathway cross-talk most likely exists between the STAT3 and PPARA as they converge on upregulation of nuclear expression of CPT1 [228,229] driving FAO as an energy source in breast cancer-adipose associations. While adiponectin signaling functionally inhibits CPT1 activity (Figure 3), and has been correlated with of inhibition of STAT3 signalling in cancer, the differential expression of adiponectin receptors in the presence of leptin may reduce this inhibition, allowing for STAT3 mediation of adipocytokine signalling and promotion of breast cancer phenotypes allowing adipose tissue to create a fostering niche for cancer to thrive [230,231,232].

## 6. Concluding Remarks

Adipose tissue plays a significant role in the development and maintenance of cancer through the formation of favourable metabolic niches. It is apparent that STAT3 acts as a metabolic modulator through which adipocytokine signaling can propagate the inflammatory signals from adipose tissue. A prime example of this is the role that adipose plays in creating a fostering environment for abberent tissues to thrive through proliferative and pro-survival signaling through IL-6 and leptin as well as through the provision of alternative energy sources in the form of free fatty acids to promote FAO in hypoxic tumour cores. Leptin signaling may lead to STAT3 activation of HIF-1α in breast cancer and therefore may also drive CPT1 expression resulting in nuclear regulation of energy generation; a critical activity for cellular survival particularly in unfavourable hypoxic environments. The specific role of STAT3 in each tumour type is determined by the metabolic requirements and the integration of multiple internal and external signals that dictate metabolism, and consequently growth, development and survival in the microenvironment (see Figure 4). Continued cytokine release into the tumour microenvironment as a result of chronic inflammation of the macroenvironment (e.g., adipose) may be detrimental to the health of normal cells but it is beneficial in tumour types requiring sustained STAT3 signalling. This further drives the argument for targeting of the niche and associated cells as opposed to the tumour when developing chemotherapeutic strategies.

## Figures and Tables

**Table 1:** Main activators of STAT3.

STAT3 Activators		Cell Lines
**Cytokines**	IL-6	Prostate cancer, pancreatic cancer, macrophages [56,57,58]
	IL-10	Chronic lymphocytic leukemia, macrophages [59]
	IL-17	Hepatocellular carcinoma, stromal cells [60]
	Interferons	Lung fibrosarcoma [61]
**Hormones**	Leptin	Hipothalamus [54]
**Growth factors**	Granulocyte colony-stimulating factor	Bone marrow neutrophils, monocytes [62,63]
	Epidermal growth factor	In vitro [64,65,66]
	Platelet-derived growth factor	3T3 cells and fibroblasts [65,67]
**Oncogenes**	Src	Fibroblasts, glioblastoma [68,69]
	Rac1	COS-1 fibroblasts [65]
	Bone marrow X-linked nonreceptor tyrosine kinase	Glioblastoma [68]
